# The association between blood MxA mRNA and long-term disease activity in early multiple sclerosis

**DOI:** 10.3389/fneur.2022.907245

**Published:** 2022-08-17

**Authors:** Eline M. E. Coerver, Eva M. M. Strijbis, Laura F. Petzold, Zoé L. E. Van Kempen, Bas Jasperse, Frederik Barkhof, Cees B. M. Oudejans, Bernard M. J. Uitdehaag, Charlotte E. Teunissen, Joep Killestein

**Affiliations:** ^1^Department of Neurology, MS Center Amsterdam, Amsterdam Neuroscience, Amsterdam UMC, Vrije Universiteit Amsterdam, Amsterdam, Netherlands; ^2^Department of Neurology, Maidstone and Tunbridge Wells NHS Trust, Kent, United Kingdom; ^3^Department of Radiology and Nuclear Medicine, MS Center Amsterdam, Amsterdam Neuroscience, Amsterdam UMC, Vrije Universiteit Amsterdam, Amsterdam, Netherlands; ^4^Centre for Medical Image Computing, Queen Square Institute of Neurology, University College London, London, United Kingdom; ^5^Molecular Biology Laboratory, Department of Clinical Chemistry, Amsterdam UMC, Vrije Universiteit Amsterdam, Amsterdam, Netherlands; ^6^Neurochemistry Laboratory and Biobank, Department of Clinical Chemistry, Amsterdam Neuroscience, Amsterdam UMC, Vrije Universiteit Amsterdam, Amsterdam, Netherlands

**Keywords:** demyelinating disease, multiple sclerosis, MRI, myxovirus resistance protein A (MxA), mRNA

## Abstract

**Background:**

Myxovirus resistance protein A (MxA) is a protein that is upregulated by interferon-beta. Homeostatic MxA mRNA levels are potentially correlated with inflammatory disease activity in multiple sclerosis (MS) and could have an important role in MS pathology.

**Aim:**

To investigate the association between myxovirus resistance protein A (MxA) mRNA levels in blood and disease activity and progression in MS over a long-term follow-up period.

**Methods:**

Baseline blood MxA mRNA levels were determined in a prospective cohort of 116 untreated patients with a clinically isolated syndrome (CIS) or early relapsing remitting MS (RRMS), and related to long-term relapses, radiological disease activity, clinical scores [Expanded Disability Status Scale (EDSS), timed-25-foot walk (T25FW), 9-hole-peg test (9HPT)], MS type, and disease modifying therapy (DMT) use.

**Results:**

Low MxA mRNA levels were associated with the occurrence of ≥9 T2-lesions on MRI imaging and the occurrence of relapses during long-term follow-up (median 11 years, IQR 5.91–13.69 years). MxA mRNA levels were not associated with EDSS, T25FW, 9HPT, and MS subtype.

**Conclusion:**

Baseline MxA mRNA levels are associated with long-term development of T2-lesions on MRI-scans in our cohort. This confirms the relevance of the endogenous interferon-beta system in the occurrence of MS disease activity.

## Introduction

The disease course of multiple sclerosis (MS) can vary greatly between patients. Inflammatory disease activity is an important determinant of the disease course in patients with relapsing forms of MS. It can be monitored by the occurrence of relapses and the presence of new or enlarging T2-lesions or contrast-enhancing lesions (CELs) on MRI (1).

Myxovirus resistance protein A (MxA) is one of the proteins specifically upregulated by interferon-beta. MxA mRNA levels are used in clinical practice to determine bioactivity of interferon-beta and its consequent effectiveness to suppress or prevent inflammatory disease activity in MS patients. Low MxA mRNA levels after interferon-beta injection indicate lack of treatment efficacy, caused by formation of neutralizing antibodies (NAbs) against interferon-beta (2–4). In addition to its function as a biomarker for bioactivity related to interferon-beta treatment, the association between spontaneous MxA mRNA level and inflammatory disease activity in MS has been investigated (5, 6). One hypothesis is that in patients with substantial inflammatory disease activity endogenous interferons are less effective, in which case it would be expected that low MxA mRNA levels are associated with an active disease course associated with increased inflammation (5, 6). In 2010 Van der Voort et al. investigated the association between homeostatic MxA mRNA levels and inflammatory disease activity in MS. Low blood MxA mRNA levels were associated with a higher number of CELs on baseline MRI, higher frequency of relapses and a shorter time to first relapse (7). To provide more insight into the role of the endogenous interferon-beta system in the severity of inflammatory disease activity in MS, it is of interest to investigate if this association between homeostatic MxA mRNA levels and clinical and radiological disease activity in MS is still present in the long-term. Therefore, we reevaluated this well-documented, prospective cohort to determine whether the association of homeostatic low MxA mRNA levels with inflammatory disease activity and disability is still present during long-term follow-up.

## Methods

### Study design and data collection

The patient cohort that was studied has been described previously (7). Patients were originally selected from a prospective cohort of patients that presented with a CIS or were diagnosed with RRMS in the 6 months before inclusion. Only patients that were not treated with disease modifying therapy (DMT) at the moment of blood collection were included. Expanded Disability Status Scale (EDSS), timed-25 foot walk test (T25FW) and 9-hole peg test (9HPT) were performed at baseline and during follow-up by experienced raters. In addition, data on MS type and DMT use over the course of the disease were collected. The average time interval between baseline and follow-up visits was 18.2 months (SD 8.1 months).

At baseline, peripheral blood was collected in PAXgene tubes, which were kept at room temperature for at least 2 h after blood collection and then frozen at −80°C. Automated RNA isolation was performed in the VU Medical Center Amsterdam on the BioRobot MDX (Qiagen) according to the instructions of the manufacturer (PaxGene Blood RNA MDx kit). MxA mRNA expression was assessed by one-step real-time quantitative RT-PCR with Taqman probes and normalized to housekeeping gene glyceraldehyde 3-phosphate dehydrogenase (GAPDH) expression level to correct for experimental variations (7, 8). To monitor for active infections, total leukocyte counts and differentiation were also determined at the moment of blood collection. MxA mRNA expression was then measured as described previously (8), and divided into two categories based on a median split.

Brain MRI including FLAIR/T2 sequences and a 2DT1-post contrast series were obtained at baseline. Baseline scans were performed on 1.0 or 1.5 T scanners as previously described (7). Follow-up scans were performed for clinical purposes according to a standardized MRI protocol that always included FLAIR and dual-echo T2 sequences. 2DT1 post contrast sequences were obtained when necessary according to clinical guidelines. The number of new or enlarging T2-lesions during any of the follow-up moments, and the presence of contrast-enhancing lesions (CELs) in case of gadolinium administration was evaluated by neuroradiologists with extensive experience with MS and related diseases. For the analyses, the total number of new T2-lesions during the follow-up period was used and divided into two categories (<9 vs. ≥9 new T2-lesions). These categories were chosen because the distinction between <9 vs. ≥9 new T2-lesions has in the past been part of the MRI-criteria for MS diagnosis, and was for that reason well-documented in our cohort (9, 10).

### Definitions

Relapses were defined as the onset of new or recurrent symptoms that last more than 24 h, that are accompanied by new objective abnormalities on a neurological examination and not explained by other non-MS causes. Radiological disease activity was defined as the presence of new T2-lesions and/or gadolinium enhanced lesions on follow-up brain MRI.

For the longitudinal analysis on clinical progression, a clinically significant change was defined as: a 20% increase on the T25FW (11), a 20% increase on the 9HPT (12), and a significant increase in EDSS was scored as a ≥1.5-point increase if baseline EDSS was 0, a ≥1-point increase if baseline EDSS was 1.0–5.0, and a ≥0.5-point increase if baseline score was ≥5.5 (13). In addition, “EDSS-plus” progression was assessed, defined as a significant worsening of EDSS, 9HPT and/or TWT during follow-up.

### Statistical analysis

Cross-sectional analyses were conducted using Mann Whitney for categorical variables and Kruskal Wallis for continuous variables. Longitudinal analyses were conducted with linear regression for continuous outcome variables and logistic regression for dichotomous and categorical outcome variables. All analyses were corrected for sex, age at baseline and, if relevant, follow-up duration, use of DMT, and the number of T2-lesions on baseline MRI. Statistical analyses were performed using IBM SPSS Statistics version 26.0.

## Results

### Baseline

In total, 116 patients were included in the analyses between November 2002 and March 2007. As previously described (7), 74 (63.8%) were female. At baseline, 67 patients (57.8%) were diagnosed with RRMS and 49 (42.2%) with a CIS, according to the standard diagnostic criteria at that time (14, 15). Median MxA mRNA level was 0.075 (IQR 0.015–0.123). Median follow-up duration was 11.05 years (IQR 5.91–13.69 years). Baseline characteristics are depicted in Table 1.

**Table 1:** Baseline characteristics.

**Baseline characteristics**	**n = 116**
Age at onset, y, mean (SD)	32.9 (9.1)
Age at baseline, y, mean (SD)	34.3 (9.3)
Sex, n (%) female	74 (63.8)
EDSS, median (IQR)	2.0 (1.5–3.0)
T25FW, median (IQR)	3.8 (3.4–4.2)
9HPT, median (IQR)	
Dominant hand	17.3 (15.3–19.1)
Non-dominant hand	18.3 (17.0–19.9)
MS subtype, *n* (%)	
Clinically isolated syndrome	49 (42.2)
Relapsing-remitting	67 (57.8)
MxA mRNA/GAPDH^*^, median (IQR)	0.08 (0.02–0.12)

*SD, standard deviation; IQR, interquartile range; EDSS, Expanded Disability Status Scale; T25FW, timed 25-foot walk test; 9HPT, 9-hole peg test; CIS, clinically isolated syndrome; RRMS, relapsing-remitting MS; MxA, Myxovirus resistance protein A; GAPDH, glyceraldehyde 3-phosphate dehydrogenase*.

* *MxA mRNA expression was normalized to the expression level of housekeeping gene GAPDH*.

All patients were treatment naive at the moment of blood collection. At the moment of blood sampling, 23 patients were experiencing a clinical relapse, whereas in other patients, blood sampling was done during remission. Previous analysis showed that MxA mRNA levels were lower in patients with a relapse at the time of blood sampling [median 0.036 (IQR 0.009–0.075)], compared to those in remission [median 0.084 (IQR 0.021–0.145)] (*p* = 0.002) (7). To exclude possible bias caused by the timing of blood collection, all analyses were done for the complete patient population (116 patients) as well as for the subgroup of patients in which blood collection was done at remission (93 patients). None of the patients reported any viral infections at the moment of blood sampling, and leukocyte counts and differentiation were normal (7).

### Follow-up

At the end of the follow-up period, 93 patients (80.2%) had a diagnosis of RRMS, 12 (10.3%) of CIS, 8 (6.9%) of secondary progressive MS (SPMS) and 3 (2.6%) of primary progressive MS (PPMS). Thirty-two patients (27.6%) converted from CIS to RRMS during follow-up. No significant association was found between baseline MxA mRNA level and MS type at follow-up, or between baseline MxA mRNA and conversion from CIS to RRMS during follow-up. Follow-up characteristics are depicted in Table 2.

**Table 2:** Follow-up characteristics.

**Follow-up characteristics**	**n = 116**
Follow-up duration	
Months, median (IQR)	132.77 (71.0–164.4)
Years, median (IQR)	11.06 (5.9–13.7)
EDSS, median (IQR)	3.0 (2.0–4.0)
T25FW, median (IQR)	4.2 (3.7–5.1)
9HPT, median (IQR)	
Dominant hand	18.9 (16.8–21.6)
Non-dominant hand	20.4 (18.0–23.3)
MS subtype, n (%)	
CIS	12 (10.3)
RRMS	93 (80.2)
SPMS	8 (6.9)
PPMS	3 (2.6)
Relapse during follow up, n (%)	
Yes	80 (69.0)
No	36 (31.0)
Radiological disease activity during follow-up, n (%)	
Yes	105 (90.5)
No	11 (9.5)

*SD, standard deviation; IQR, interquartile range; EDSS, Expanded Disability Status Scale; T25FW, timed 25-foot walk test; 9HPT, 9-hole peg test; CIS, clinically isolated syndrome; RRMS, relapsing-remitting MS; SPMS, secondary progressive MS; PPMS, primary progressive MS*.

#### Relapses

**Figure 1:**
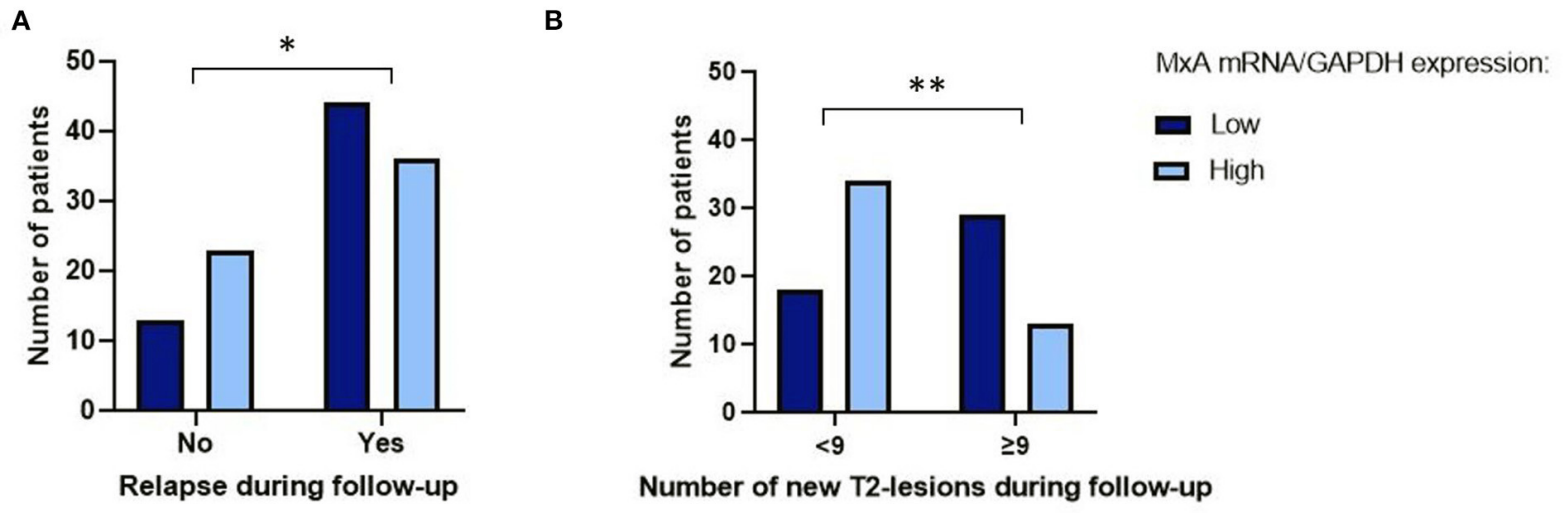
Baseline MxA mRNA level vs. occurrence of relapses and new T2-lesions on MRI during follow-up. **(A)** Number of patients with low vs. high MxA mRNA/GAPDH expression that experience at least one relapse during follow-up. *Results of logistic regression analysis: B = −0.81, Exp(B) = 0.45, *p* = 0.070, 95% CI 0.19–1.07. **(B)** Number of patients with low vs. high MxA mRNA/GAPDH expression that have <9 vs. ≥9 new T2-lesions on MRI during follow-up. **Results of logistic regression analysis: B = −1.86, Exp(B) = 0.16, *p* = 0.012, 95% CI 0.04–0.66. All patients included. GAPDH, glyceraldehyde 3-phosphate dehydrogenase.

Eighty patients experienced at least one relapse during follow-up. Median number of relapses during follow-up was 1 (IQR 0–3). A baseline MxA mRNA level of <0.075 was associated with the occurrence of at least one relapse during follow-up, although not statistically significant in the complete patient group [B = −0.81, Exp(B) = 0.45, *p* = 0.070, 95% CI 0.19–1.07] (see also Figure 1). When repeated in only the patients that were in remission during blood collection, this effect was statistically significant [B = −1.18, Exp(B) = 0.31, *p* = 0.025, 95%CI 0.11–0.86]. In addition, low baseline MxA mRNA level (<0.075) was associated with a higher number of relapses during follow-up [complete patient group: U = 1295, Z = −2.18, *p* = 0.029, remission group only: U = 831, Z = −1.83, *p* = 0.068]. No significant association was found between baseline MxA mRNA level and time to first relapse.

#### Clinical scores

Median EDSS at follow-up was 3.0 (IQR 2.0–4.0). During follow-up, 47.7% of patients experienced a significant increase in EDSS. No significant association was found between baseline MxA mRNA level and the occurrence of a significant increase in EDSS during follow-up [B = −0.16, Exp(B) = 0.85, *p* = 0.678, 95% CI 0.40–1.82]. In addition, no significant association was found between baseline MxA mRNA level and the occurrence of a significant increase in T25FW [B = 0.50, Exp(B) = 1.65, *p* = 0.241, 95 CI 0.72–3.79] or 9HPT score [dominant hand: B = 0.01, Exp(B) = 1.0, *p* = 0.998, 95% CI 0.05–20.15, non-dominant hand: B = 0.12, Exp(B) = 1.13, *p* = 0.929, 95% CI 0.09–14.94] during follow-up. Also, no significant association was found between baseline MxA mRNA level and “EDSS-plus” worsening during follow-up [B = 0.02, Exp (B) = 1.02, *p* = 0.965, 95% CI 0.47–2.19]. All analyses were repeated in the remission group only, which also showed no significant association between these clinical scores and baseline MxA mRNA level.

#### DMT use

Sixty-three patients (54.3%) started using DMT at any moment during follow-up. Fifty-two patients (44.8%) did not; of one patient this was unknown. At the end of the follow-up period, 42 patients (33.6%) used first-line DMT (any of the interferons, glatiramer acetate, teriflunomide, dimethylfumarate) and 16 patients (13.8%) used second-line DMT (natalizumab, fingolimod, ocrelizumab). Baseline MxA mRNA level was not associated with the start of DMT during follow-up, with the type of DMT used at follow-up, or with the use of first line or second line DMT during follow-up.

#### MRI parameters

Median number of MRI-scans done during 11 years of follow-up was 6 per patient (IQR 4–10). Ninety percent of patients experienced MRI-activity during follow-up, meaning any new T2-lesions or CELs on any MRI scan available during follow-up. A baseline MxA mRNA level of <0.075 was associated with a cumulative number of ≥9 new T2-lesions during follow-up, compared to <9 new T2-lesions during follow-up. This association was present when including all patients in the analysis [B = −1.86, Exp(B) = 0.16, *p* = 0.012, 95% CI 0.04–0.66] (see also Figure 1). In the remission group, the same effect was seen, although not statistically significant [B = −1.51, Exp(B) = 0.22, *p* = 0.079, 95% CI 0.04–1.19]. No significant association was found between baseline MxA mRNA level and time to first MRI activity.

## Discussion

In our prospective cohort of CIS and early RRMS patients we found low spontaneous MxA mRNA levels in the first months after diagnosis to be associated with the occurrence of a larger number of new T2 lesions during a median follow-up period of 11 years. Low baseline MxA mRNA levels were also associated with the occurrence, and a higher number of, relapses during follow-up. No significant association was found between spontaneous MxA mRNA level and clinical scores (EDSS, T25FW, 9HPT) and MS type at follow-up.

MxA mRNA and MxA protein are present at stable low levels in blood under normal circumstances, and are rapidly upregulated by interferon type I in a dose-dependent manner. They are therefore known as reliable markers for interferon type I responsiveness (16). Endogenous interferon type I pathways play various important roles in the human immune system. For example, a dysregulation of type I interferon pathways is found in various chronic inflammatory autoimmune diseases, such as Sjögren's syndrome and systemic lupus erythematosus (SLE) (17–19).

In MS, an increased activation of type I interferon pathways has been associated with an upregulation of immunoregulatory cytokines and reduced T-cell responses, which might be associated with a dampening of inflammatory disease activity (20–22). Therefore, it is conceivable that in patients with a more active inflammatory disease course, characterized by the occurrence of (more) relapses and new T2-lesions and/or enhancing lesions on MRI, endogenous interferon-beta pathways are insufficiently capable to upregulate and protect against inflammatory activity, as reflected by low blood levels of MxA mRNA in these patients.

Interesting in this regard is the growing evidence of the role of viral infections in the pathogenesis of MS. One of the main functions of type I interferon system is its antiviral function, and people with a defective interferon type I system are likely more susceptible to viral infections or a more severe course of infectious disease (23, 24). Regarding MxA, it is known that MxA proteins, amongst many other proteins, also possess antiviral properties: in case of certain viral infections, such as influenza and measles, MxA mRNA and MxA protein are rapidly upregulated by endogenous interferon type I and play a role in the inhibition of multiplication of these viruses (16, 25). An impairment in interferon type I pathways may lead to an impaired upregulation of MxA mRNA, resulting in lower MxA mRNA and protein levels.

Multiple studies have shown a potential triggering effect of viral infections in the development of MS, especially Epstein-Bar virus (EBV) infections, of which increasing evidence is found for a major causal role in MS pathogenesis (26, 27). The altered interferon type I system that is found in MS could possibly be related to this phenomenon, considering the importance of the type I interferon system in viral immunity, such as immunity against EBV infections (20–22, 28, 29). This alteration of the interferon type I system might be reflected by a change in MxA mRNA level.

In 2010, we described the association between spontaneous MxA mRNA levels and disease activity with a median follow-up period of 44 months (8). There was a lower relapse rate and a longer time to new relapses in patients with high spontaneous MxA mRNA levels. Even though there was no significant association between MxA mRNA level and annualized number of new T2-lesions or the occurrence of CELs during follow-up, the proportion of patients with no or low number of T2-lesions after a follow-up period of 1 year was higher in patients with a high spontaneous MxA mRNA level at baseline. The current study extends these findings by confirming the association between MxA mRNA levels and clinical and radiological disease activity over a follow-up period of 11 years.

It must be noted that this is an observational study in a real-world setting. The decisions on follow-up and treatment were made based on the clinical treatment protocols at that moment. At the time of treatment initiation, interferon and glatiramer acetate were the only available treatment options in CIS or early MS. During the period of follow-up, higher efficacy compounds were admitted to the market (e.g., natalizumab, fingolimod, ocrelizumab), resulting in a change in the treatment landscape of MS. Additionally, not all MRI-scans were made with gadolinium contrast administered during follow-up.

Despite these limitations, it would be of interest to validate the results found in this study in a larger patient group. Our study suggests that an impairment in the type I interferon system could play an important role in inflammatory MS pathology, as reflected by low MxA mRNA levels, which are associated with long-term inflammatory disease activity in our study. In addition to providing more insight into the mechanisms of inflammatory disease activity in MS, homeostatic MxA mRNA level could also be of interest as an easy-to-use prognostic biomarker for long-term inflammatory disease activity in MS. Currently, the best prognostic factors for long-term disease activity are MRI measurements. Number of active lesions on brain MRI and spinal cord lesions on MRI are known to be associated with disability in MS. In addition to using MRI, the development of easy-to-use biomarkers that predict inflammatory disease activity would greatly benefit clinical decision making regarding treatment of MS patients and improve personalized patient care. For example, if the occurrence of long-term inflammatory disease activity can be predicted easily and precisely, treatment decisions can be adjusted based on this knowledge, such as the decision when to start with DMT, and which DMT should be started.

In conclusion, our long-term clinical and radiological follow-up data suggest an important mechanistic effect of the endogenous type-1 interferon system reflected in MxA mRNA in the expression of inflammatory pathology of MS. If confirmed in other populations, MxA mRNA could also be an interesting candidate as prognostic biomarker for long-term inflammatory disease activity in MS.

## Data availability statement

The raw data supporting the conclusions of this article will be made available by the authors, without undue reservation.

## Ethics statement

The studies involving human participants were reviewed and approved by Medical Ethical Committee of the Amsterdam UMC, location VUmc. The patients/participants provided their written informed consent to participate in this study.

## Author contributions

EC, ES, and JK contributed to conception and design of the study, analysis and interpretation of data, and drafted the manuscript. LP, ZV, BU, FB, CO, BU, and CT contributed to the interpretation of data and revision of the manuscript for intellectual content. All authors contributed to the article and approved the submitted version.

## Funding

EC and ES received funding from ZonMW, Grant Number 848043001, and Stichting MS Research, Grant Number 17-992. FB is supported by the NIHR Biomedical Research Centre at UCLH.

## Conflict of interest

LP received funding for courses from Novartis and Biogen. FB acts as a consultant to Biogen-Idec, Janssen Alzheimer Immunotherapy, Bayer-Schering, Merck-Serono, Roche, Novartis, Genzyme, and Sanofi-aventis. BU reports personal fees from Genzyme, Biogen Idec, Teva Pharmaceutical Industries, Merck Serono, and Roche. CT has served on advisory boards for Roche, has received non-financial support in the form of research consumables from ADx NeuroSciences and Euroimmun, and has performed contract research or received grants from Probiodrug, Biogen, Esai, Toyama, Janssen Prevention Center, Boehringer, Axon Neuroscience, EIP Pharma, PeopleBio, and Roche. JK has accepted speaker and consulting fees from Merck, Biogen, Roche, Teva, Genzyme, and Novartis. The remaining authors declare that the research was conducted in the absence of any commercial or financial relationships that could be construed as a potential conflict of interest.

## Publisher's note

All claims expressed in this article are solely those of the authors and do not necessarily represent those of their affiliated organizations, or those of the publisher, the editors and the reviewers. Any product that may be evaluated in this article, or claim that may be made by its manufacturer, is not guaranteed or endorsed by the publisher.
